# An Analysis of Differences in the Role of Friendships and the School Class in Children’s Perceptions of *Bullying* in England and *Ijime* in Japan: A Translation and Expansion of Kanetsuna (2009)

**DOI:** 10.3390/ijerph18158234

**Published:** 2021-08-03

**Authors:** Tomoyuki Kanetsuna, Peter K. Smith

**Affiliations:** 1 Faculty of Education, Kagawa University, Kagawa 7608522, Japan; 2 Unit for School and Family Studies, Department of Psychology, Goldsmiths, University of London, London SE14 6NW, UK; p.smith@gold.ac.uk

**Keywords:** bullying, *ijime*, perception, friendships

## Abstract

This is an English translation with some expansion of the article originally published in Japanese as a university bulletin in 2009. Previous research has found both differences and similarities between *ijime* in Japan and *bullying* in England. *Bullying* is often by pupils in different classes or higher year groups whom the victim does not know very well; *ijime* is often by victims’ classmates whom the victim knows very well. However, it has not been shown whether these differences are found for all types of bullying, or how they relate to friendships generally and the impact of differing school systems. We aimed to see whether previously found differences between *ijime* and *bullying* could be replicated, and, if so, whether they held for six different types of victimization, and whether friendship characteristics were consistent with explaining why they occur. To investigate the role of friendships and their location, 1036 Japanese and 931 English secondary school pupils participated in a comparative study of perceptions of *bullying* and *ijime*. The previous differences were confirmed and found to hold irrespective of type of bullying. Japanese pupils mainly formed friendships on a class basis, English pupils on a broader basis including pupils in different years. In school, English pupils spent much time in the playground with their friends and saw this as a likely venue for *bullying*, whereas Japanese pupils spent more time in the classroom and saw this as a likely venue for *ijime*. The difference in friendship formation, together with differences in the organization of class-based teaching in the two countries, are hypothesized to play a significant role in explaining some differences between *bullying* and *ijime*.

## 1. The Role of Friendships and the School Class in Children’s Perceptions of *Bullying* in England and *Ijime* in Japan: A Translation and Expansion of Kanetsuna (2009)

The concept of *bullying* is generally defined in western cultures as an aggressive behavior characterized by repetition of actions and asymmetric power relationships [1]. However, the definition and understanding of bullying-like phenomena varies by culture [2]. An interesting case is the comparison between *bullying* in western countries, and *ijime* in Japan. *Ijime* is the Japanese term considered most similar to *bullying* and has a research tradition spanning 40 years [3]. Morita defined *ijime* as “a type of aggressive behavior by which someone who holds a dominant position in a group-interaction process, by intentional or collective acts, causes mental and/or physical suffering to another inside a group” [3].

The first systematic comparison between *bullying* in England and *ijime* in Japan, conducted as part of a cross-national study also including Norway and the Netherlands, was carried out in 1998. This found much similarity between *bullying* and *ijime* in overall prevalence, major types, gender differences and age trends [4]. However, Morita and his colleagues argued that, while similar to *bullying*, *ijime* is more weighted towards verbal and indirect, rather than physical, aggression, and more often causes victims psychological suffering. It is more characteristic of within-grade relationships, rather than of an older pupil bullying a younger one [3]. In order to verify these arguments, Kanetsuna and colleagues conducted more detailed direct comparisons between pupils in England and Japan using one-to-one in depth structured interviews and self-report questionnaire surveys with pupils [5,6,7]. They identified a number of differences in children’s understanding of *bullying* compared to *ijime*. *Bullying* in England was commonly understood as often being carried out in the playground, as a form of direct physical and verbal aggression, by physically big and strong person(s) who may be older than the victims and whom the victims may know but not in a friendly way, or not know very well or at all. *Ijime* in Japan, in contrast, was commonly understood as often being carried out in the classroom by direct verbal as well as indirect social or relational means by the victims’ classmates, or at least pupils in the same year group whom the victims know very well.

### 1.1. Different Forms of Bullying/Ijime

These findings by Kanetsuna and colleagues can be seen as children’s general perceptions and understanding of the nature of *bullying*/*ijime*, and, therefore, are likely to be a reflection of the most characteristic situation in each country. However, there are several different forms of behavior that can be regarded as *bullying* or *ijime*, including physical, verbal, rumor spreading, and social exclusion. Pupils in Japan and England have different ideas of how they cope with such different forms of aggression [5,7]. It is important to examine their perceptions and understanding about each different form of *bullying*/*ijime* behavior individually. For instance, friendship characteristics might influence experiences of physical bullying, or of social exclusion, differently in the two countries.

### 1.2. Friendship Formation

Given the friendship and location differences in English and Japanese pupils’ perceptions of *bullying* and *ijime*, it is important to gather evidence on who children in each country form their friendships with, and where they most often interact with them. Morita and Kiyonaga suggested that, in Japanese schools, the social group is more likely to be formed within a class, and pupils have fewer interactions with pupils in different classes or year groups compared to pupils in England [8]; however, this supposition lacks direct empirical support. If true, then the aggressor(s) and victims of *ijime* will be more likely to share the same social group, and pupils will be more likely to experience indirect forms of *ijime* in the classroom by their classmates or even by their ”friends”; whereas, in England, pupils may have more interactions with pupils in different classes and year groups, bullies and victims will be less likely to share the same peer group, and pupils will be more likely to experience direct forms of *bullying* in the playground by older or unknown peers. Relatedly, it would be important to know how much time pupils spend with friends in different places in school; if pupils in Japan spend more time with peers in the classroom and pupils in England spend more time with peers in the playground, it seems natural to see more *ijime* in the classroom and more *bullying* in the playground, whatever type of *bullying*/*ijime* it is.

Morita et al. [3] argued that some forms of indirect *bullying*/*ijime* such as ignoring and social exclusion should be more effective if the aggressor(s) and the victim belong to the same social group. Therefore, these types of *bullying*/*ijime* could be more likely to occur between pupils who know each other very well and could be more likely to happen in closed places like the classroom, rather than in the playground. Direct physical or verbal forms of *bullying*/*ijime*, on the other hand, should be more likely from someone physically stronger than the victim, and, unlike indirect forms, such direct physical or verbal forms of *bullying*/*ijime* can still be very effective if the victim has no knowledge about the aggressor(s). Additionally, these direct forms of *bullying*/*ijime* are more visible, making it easy for teachers and other pupils to intervene. Therefore, places with less adult supervision, such as the playground in schools in England, could be a more likely place for this to happen.

### 1.3. Aims of the Study

We had three aims in this study:

To test whether previous findings about differences between *bullying* and *ijime* can be replicated, as regards whether bully–victim relationships are mainly within or across classes and year groups, and mainly in the classroom or the playground;

To see if such differences are found consistently for six different types of victimization

To gather data on the extent to which friendships are within or between classes and year groups in England and Japan, and where friends most commonly interact, to see whether this is consistent with explaining the differences found.

We examined the above issues by using similar methodologies with secondary school pupils in England and Japan. We examined perceptions of six different forms of *bullying*/*ijime* behavior; we did not give a specific definition of either *bullying* or *ijime*, but used six hypothetical scenarios. We examined where they were likely to happen, and the likely relationship of the aggressor(s) with the victim. We also sought information on friendships in the same class, different classes, and different year groups, and how much time was spent with these friends in different places.

## 2. Method

### 2.1. Participants

A convenience sample of 1036 Japanese pupils (520 males, 516 females) from four lower-secondary schools in Chiba, an urban district on the outskirts of Tokyo, and 931 English pupils (468 males, 463 females) from five secondary schools in London, Kent, and Leicester participated in the anonymous self-report questionnaire study conducted during 2003. Pupils were drawn from years 8, 9, 10, and 11 in the English schools (due to smaller numbers, years 10 and 11 were combined), and equivalent grades in Japanese schools. The numbers of participants in each year group were: Japan: year eight, 452 (224 males, 228 females); year nine, 275 (151 males, 124 females); year ten, 309 (145 males, 164 females). England: year eight, 324 (170 males, 154 females); year nine, 465 (237 males, 228 females); years ten/eleven, 142 (61 males, 81 females). The mean age of the Japanese sample was 13.4 years (SD = 0.97) and of the English sample, 13.2 years (SD = 0.97). All schools were state funded and non-residential. Given differences between Japan and England in economic structure and disparity of wealth, equating measures of socio-economic level is difficult, but none of the schools sampled either particularly rich families or areas of social deprivation, and were considered reasonably typical of schools in each country.

### 2.2. Measures

The questionnaire was designed in English and piloted extensively with 12–14-year-old pupils in England to examine whether each question was clear enough to understand and the time taken to complete the questionnaire. As a result, it was slightly shortened; the final version of the questionnaire took approximately thirty minutes to complete. The English version was translated by the first author into Japanese. The Japanese version was then back-translated into English by another Japanese-English bilingual person and was checked by an English bilingual person to ensure the Japanese version had been reliably translated.

The questionnaire contained two separate sections. Following demographic information, Section 1 asked about friendships of three kinds: (1) How many good friends do you have in your class/in different classes but in the same year group/in different year groups? (Pupils entered actual numbers), and, then, for each of the three kinds, in six places: (2) How often do you spend time with these friends during break-times in classroom/break-times in friends’ classroom/break-times in playground/break-times in other places in school (corridors, stairs, library, gym, other rooms)/on the way to school and to home/outside school? (5-point scale: 1: Never; 2: Rarely [once a week]; 3: Sometimes [twice or three times a week]; 4: Often [three or more times a week]; and 5: Almost always [every occasion]).

Section 2 was on general understanding of six different forms of *bullying*/*ijime* behavior (physical, verbal, ignoring, social exclusion, stealing/hiding/taking money or belongings, and malicious rumor spreading/nasty note-sending). Previously [7], we had used four different forms of behavior: physical, verbal, ignoring/social exclusion, and rumor spreading/note-sending; however, in this study, we treated ignoring and social exclusion as two different behaviors, since social exclusion is a group act while ignoring can be at an individual level as well. We also added stealing/hiding/taking money or belongings as another form of *bullying*/*ijime* behavior. Although cyber forms of aggression have become recognized as new types of *bullying*/*ijime* in both countries in more recent years [9,10], these were not included in this study since they were not frequent at the time (2003).

Pupils were given six different hypothetical scenarios:

Physical: Imagine one student or a group of students hit, kick, and punch another student who cannot fight back or defend him/herself effectively.

Verbal: Imagine one student or a group of students say mean or unpleasant things to another student, make fun of him/her, or call him/her mean and hurtful names.

Ignoring: Imagine one student or a group of students refuse any sort of communication with one student as if he/she does not exist or is invisible.

Social exclusion: Imagine a group of students actively try to exclude one student from their group of friends, tell him/her “No, We don’t want to play with you”.

Stealing/hiding/taking money or belongings: Imagine one student or a group of students hide, break, steal, or take another student’s money or valuable belongings.

Malicious rumor spreading/nasty note-sending: Imagine one student or a group of students spread nasty rumors about another student, talk behind his/her back, or gossip about him/her.

For each scenario, pupils were asked the following questions:

“How likely do you think this situation is to happen in the classroom/playground/elsewhere in school/on the way to home or to school/outside school?”

“How likely do you think the aggressor is to be classmates/person or people in different class but in the same year group/person or people in higher year group?”

“How likely do you think the relationship between aggressor(s) and the victim is to be friends/they know each other but not in a friendly way/they don’t know each other very well or at all?”

All were answered on 5-point scales: 1: Not likely; 2: Slightly likely; 3: Quite likely; 4: Likely; 5: Very likely.

### 2.3. Procedural and Ethical Issues

In both countries, a convenience sample was used. The head teachers of schools in the area were approached by telephone and email, given a description of the study, and asked if they would permit their pupils to take part. For those schools from which agreement was given, meetings with members of staff were held in order to discuss the study further. Schools were given an opportunity to examine the questionnaire and asked whether they would like the researcher to make any changes in the questionnaire or to omit particular questions (no changes were actually requested by any schools).

In the case of both English and Japanese versions of the questionnaire, participants were given general information about anonymity of data and their right not to take part in the study and not to answer any question that they felt uncomfortable with even after deciding to take part. The study was approved by the institutional ethics committee.

Questionnaires were delivered by the first author to each school, given on a class basis by members of staff, and collected. Each school was given written feedback about the findings, with an overall summary, and detailed results for that school. However, individual responses were not given to any school due to the anonymity of the data collection procedure.

### 2.4. Analysis Plan

Statistical analysis was conducted using SPSS. We used MANOVA with factors of nationality (2 levels), gender (2), and year groups (3), and ANOVA as a follow-up analysis for main effects of each factor and their interactions. Here, we report on nationality differences. Details of main effects of gender and year group, and interactions, can be obtained from the first author or in Kanetsuna (2004) [6]. Due to a large number of tests being made, only results found significant at the *p* < 0.01 level are considered.

## 3. Results

### 3.1. The Perceived Nature of Bullying/Ijime

Likely places to happen: Pupils were asked how likely they thought each scenario would happen in the classroom, playground, elsewhere in school, on the way to school/to home, and outside school. Mean scores and main effects of nationality are shown in Table 1.

There were significant main effects of nationality for all six scenarios. The follow-up analysis revealed that Japanese pupils rated the classroom much higher, and English pupils rated the playground much higher, for all six scenarios. English pupils also rated all the other places higher for all the scenarios, with the exception of elsewhere in school for ignoring and rumor spreading/note-sending (see Table 1).

Likely aggressor(s): Pupils were asked how likely they thought the aggressor(s) of each scenario would be classmates of the victim, pupils in different class but the same year group, and pupils in higher year group. Mean scores and the main effect of nationality are shown in Table 2.

There were significant main effects of nationality for all six scenarios. Follow-up analyses revealed that, for all six scenarios, Japanese pupils rated pupils in the same class higher than English pupils, and English pupils rated pupils in higher years higher than Japanese pupils. Japanese pupils rated pupils in different classes but the same year group higher for all scenarios except physical aggression (Table 2).

Likely relationships between aggressor(s) and the victim: Pupils were asked how likely they thought the aggressor(s) and victim of each scenario would be friends with each other, know each other but not friends, and not know each other very well or at all. Mean scores and the main effects of Nationality are shown in Table 3. There were significant main effects of nationality for all six scenarios. The follow-up analyses revealed that, for all six scenarios, Japanese pupils rated friends with each other higher than English pupils, and English pupils rated not know each other very well or at all higher than Japanese pupils. There was less difference by nationality for know each other but not friends, but it was rated higher by English pupils for physical aggression (Table 3).

### 3.2. Relationships with Friends

Number of friends: The mean number of good friends in the same class, in different classes in the same year group, and in different year groups is shown in Table 4. Overall, the total mean number of friends was very similar between Japan (20.61) and England (19.90) (*t*_(1585)_ = 1.38, n.s.); however, who these friends were differed. There was a significant main effect of nationality for all three measures. A follow-up analysis revealed that Japanese pupils had significantly more friends than English pupils in the same class and different classes in the same year group, whereas English pupils had significantly more friends in different year groups than Japanese pupils (Table 4).

Places where pupils spent time with friends: Mean scores and main effects of nationality for the time spent with friends in the same class, friends in different classes but the same year group, and friends in different year groups for the two national samples are shown in Table 5. There were significant main effects of nationality for the time spent with all three categories of friends (Table 5). Regarding friends in the same class, Japanese pupils spent more time with friends than English pupils in the classroom, whereas English pupils spent much more time in the playground and more time in other places in school and outside school. In terms of friends in a different class but the same year group, English pupils spent more time than Japanese in the classroom, friends’ classrooms, and especially in the playground, whereas Japanese pupils spent more time on their way to school/to home. Regarding friends in different year groups, English pupils spent significantly more time than Japanese pupils at every possible place: own classroom, friend’s classroom, playground, other places in school, on the way to school/to home, and outside school.

## 4. Discussion

This study aimed, firstly, to examine and compare pupils’ perceptions and understanding of the nature of different forms of *bullying*/*ijime* and its related themes. We were able to replicate findings from previous research [5,7] regarding both common features and significant differences in the responses of pupils in England and Japan.

Regarding the general perceptions and understanding of the nature of the phenomenon, consistent with previous studies, *bullying* in England was perceived most likely to be conducted in the playground, either by pupils in different classes in the same year group or in higher year groups. They are by no means friends of the victim; instead, it is more likely that bullies and victims may know each other but are not friends with each other or they do not know each other very well or at all. *Ijime* in Japan, on the other hand, was perceived most likely to be conducted in the classroom by the victim’s “classmates” or pupils who are “in different classes but the same year group”. The aggressor(s) and the victim were perceived most likely to be “friends” with each other.

Our second aim was to examine whether these differences between *bullying* and *ijime* were consistent for all six different forms of behavior. This was very consistently found to be the case, even though effect sizes did vary (Table 1 and Table 2).

Our third aim was to examine whether these differences in pupils’ perceptions of *bullying*/*ijime* may partly stem from differences in friendships at school. Although both English and Japanese pupils reported having a considerable number of friends in the same year group, Japanese pupils had significantly more friends in the same year group, both in the same class and in different classes, whereas English pupils had significantly more friends in different year groups. Furthermore, Japanese pupils mostly spent time with friends in the same class in their own classroom and with those in different classes outside school, whereas English pupils, besides the classroom, spent most of their time with friends in the same class and in different classes in the playground. Thus, while English pupils spend considerable time with friends in different year groups in the playground, Japanese pupils seem to have few friends in different year groups (Table 3) and spend much less time with them in and outside school (Table 4).

This difference in friendship formation between pupils in England and Japan makes it understandable that *bullying* in England is often found to take place in the playground, and that *ijime* in Japan is found to take place most often in the classroom. An important question to ask is why such differences in friendships and places of friendship occur, and how it might affect the differences between *bullying* and *ijime*. One explanation could be the different school systems of each country.

Almost all state Japanese lower-secondary schools use a class system in which all pupils are allocated to one of the classes at the beginning of the year (this usually lasts at least one academic year), and they take most lessons on this class basis in their own classrooms. Class-teachers are allocated to each class to organize the class and to supervise children who belong to their class. This class system provides cohesiveness of the class and fosters close relationships between pupils who belong to the same class, and possibly between pupils and the class-teacher. However, it could also make the classroom a much more closed system, where pupils have less opportunity to form friendships with pupils in other classes or in other year groups.

This class system appears to play a significant role in characterizing the *ijime* problem in Japan as covert and indirect in nature. Morita and Kiyonaga [8] argued that, in such a closed environment, pupils as well as teachers tend to create unique characteristics or climate in their class, and if a pupil finds it difficult to fit in, s/he could easily be at great risk of isolation in the classroom and of becoming a target of *ijime*. They described *ijime* as “the interaction process of homogeneity among children”, in which a child labeled as heterogeneous in any way will either be excluded or forced, in a threatening manner, to become homogeneous to others [8].

In such an environment, the reluctance of the victim to seek external help would also be strengthened due to the difficulty in finding external help and the fear of on-going *ijime* getting worse [11]. Furthermore, the reluctance of other members of the class to intervene in the situation or to inform the class-teacher will be strengthened since such *ijime* behavior often quickly spreads to the whole classroom, and becomes a climate of the class [12]. At this stage, other non-involved members of the class find themselves under pressure to choose which side they stand by. The answer is most likely to be the aggressor(s) so as for them to defend themselves and avoid being on the wrong side of the aggressors [8].

From this point of view, the different forms of *ijime*, such as direct physical or verbal, or indirect social or relational aggression, can be regarded as merely a means of exclusion of “the heterogeneous” so as to keep the class a more desirable place for the majority. This can be one explanation why the perceived prevalence of different forms of *bullying*/*ijime* was quite similar between two countries, yet the general understanding of the nature of *bullying* and *ijime* was quite different. In other words, it seems the background intentions to *bullying* or doing *ijime* could be more important than the means of carrying it out.

In contrast to the situation in Japan, most secondary schools in England adopt the subject-teacher system in which pupils have specialist subject-teachers and different classrooms for different lessons, and, more importantly, many schools also have a system of “streaming” (class allocation based on pupils’ overall ability) or of “setting” (class allocation for individual subject based on pupils’ ability). Most state schools also have a wide range of optional subjects that pupils can choose to take depending on their interests and future plans. In other words, in state secondary schools in England, the class is not such a stable peer environment for children; instead, pupils often move from one class to another class according to their interests as well as ability for a particular subject [13]. In addition, English pupils, compared to Japanese, spend more time in the playground where pupils of all age groups are playing. Therefore, pupils in England may have more opportunity to mix with pupils in different classes as well as in different year groups and to form friendships with wider populations. In such an environment, direct physical and verbal bullying (rather than indirect social or relational) may be more likely to happen.

As Morita et al. [3] argued, indirect forms such as ignoring and social exclusion may not be effective unless the victim and the aggressor(s) belong to the same social group, and unless it is conducted in a moderately closed place, such as the classroom in Japanese schools, where pupils find it difficult to seek help from either in or outside the classroom. In contrast, direct physical and verbal forms of bullying are still effective even if the aggressor(s) and the victim do not share the same social group and have no prior relationship with each other.

While the differences between *bullying* in England and *ijime* in Japan may not be fully explained by such differences of school systems and of pupils’ friendship formations alone, they seem to be key elements. It suggests that the *ijime* problem in Japan may not be preventable with some of the strategies commonly used in England [14], such as whole-school policies, playground upgrading, or training of lunchtime supervisors. Instead, in addition to these whole-school and individual-based methodologies, class-based interventions may be more critical for successful interventions in Japanese schools [15].

How individuals perceive their friendships within such a group also seems important and needs to be examined further. Assuming that *ijime* is more often conducted within a group by one of its members (i.e., by the classmates in the classroom), pupils may form much more intimate relationships within the group where children shift their identity as an individual to one of the members of the group. In other words, once individuals form some kind of group, each individual is more likely to lose their individual identity and form a new identity as a group member. If this is the case in school classes in Japan, once an individual has been excluded from the group, the person would lose or would feel they had lost his/her identity as a whole, and that is probably what Japanese pupils find most difficult to cope with. In England, pupils do form peer groups, but these may be more open types of relationships. They may even form several different social groups with different people. In such an environment, an individual person may still identify him/herself as an individual; therefore, even if an individual was ignored or socially excluded in one group, they would still be able to join in another group. This may explain why pupils in England consider some forms of indirect aggression such as ignoring and social exclusion to be less severe or serious forms of *bullying* compared to more direct physical or verbal forms.

We should note some limitations of this study. First, we examined how pupils perceive and understand the nature of *bullying/ijime*, but we do not know from our data whether these perceptions and understandings were based on their own experiences of involving *bullying*/*ijime* situation or were based on what they had been taught at school, or possibly on what was considered socially desirable as a response. While several schools were sampled in each country, generalization of country differences must remain tentative. Furthermore, we relied on self-report data; it would be useful to supplement this with peer nominations. However, peer nomination procedure is usually not acceptable in schools in Japan, for ethical reasons. Finally, the data was gathered in 2003, and, thus, forms of cyberbullying were not included in the study. As Smith and Berkkun [16] argue, it is important to contextualize empirical data with the date of data gathering, but this should not diminish the value of the data. However, the findings do refer to that period. There have not been major changes in school organization in either England or Japan since 2003, but at that time cyberbullying was scarcely noticed. Indeed, it would be interesting to compare the findings reported here with those carried out in the future (perhaps after the COVID-19 pandemic) to ascertain how cyberbullying might affect the findings.

## 5. Conclusions

This study provides important information about the differences between *bullying* and *ijime*, and possible explanations for them. Secondary school pupils in both countries were found to have definite ideas about the nature of *bullying*/*ijime*, and their perceptions were consistent for all six different forms considered. Compared to English pupils who formed their friendships among broader populations and spent a lot of time with them in the playground, Japanese pupils formed their friendships on the basis of the class they belonged to and spent most time with them in the classroom. This seems to influence the nature of *bullying*/*ijime*. We hypothesize that the different organization of classrooms in the two countries, together with issues around group identity in Japanese pupils, lie behind much of the differences found between *bullying* and *ijime*.

## Figures and Tables

**Table 1:** Main effects of nationality on likely places for different forms of bullying/ijime.

1. Places	2. Means		3. *F* Value (*df* = 11,965)	4. *p* Value	5. Effect Size (ŋ*_p_*^2^)
	English	Japanese			
Physical aggression [Wilks’ Lambda = 0.382, *F*(5, 1951) = 632.06, *p* < 0.001, ŋ*_p_*^2^ = 0.618]					
Classroom	2.16	3.21	350.88	0.001	0.152
Playground	4.05	1.86	2009.98	0.001	0.543
Elsewhere in school	3.38	3.10	33.05	0.001	0.017
On the way to school/to home	3.44	2.37	314.54	0.001	0.139
Outside school	3.74	2.51	406.92	0.001	0.172
Verbal aggression [Wilks’ Lambda = 0.442, *F*(5, 1951) = 493.21, *p* < 0.001, ŋ*_p_*^2^ = 0.558]					
Classroom	2.53	3.91	589.11	0.001	0.232
Playground	3.87	2.07	1209.04	0.001	0.382
Elsewhere in school	3.38	3.15	22.12	0.001	0.011
On the way to school/to home	3.35	2.65	128.91	0.001	0.062
Outside school	3.56	2.47	304.46	0.001	0.135
Ignoring [Wilks’ Lambda = 0.545, *F*(5, 1951) = 325.26, *p* < 0.001, ŋ*_p_*^2^ = 0.455]					
Classroom	2.57	4.06	714.42	0.001	0.268
Playground	3.56	2.29	445.34	0.001	0.186
Elsewhere in school	3.23	3.16	1.13	Not sig.	0.001
On the way to school/to home	3.14	2.68	53.10	0.001	0.026
Outside school	3.26	2.44	164.21	0.001	0.077
Social exclusion [Wilks’ Lambda = 0.653, *F*(5, 1951) = 207.41, *p* < 0.001, ŋ*_p_*^2^ = 0.347]					
Classroom	2.41	3.53	394.68	0.001	0.168
Playground	3.65	2.56	320.02	0.001	0.141
Elsewhere in school	3.26	2.99	23.84	0.001	0.012
On the way to school/to home	3.03	2.57	66.12	0.001	0.033
Outside school	3.26	2.74	75.60	0.001	0.037
Stealing/hiding/taking money or belongings [Wilks’ Lambda = 0.609, *F*(5, 1951) = 251.02, *p* < 0.001, ŋ*_p_*^2^ = 0.391]					
Classroom	2.64	3.73	327.94	0.001	0.144
Playground	3.36	1.91	695.40	0.001	0.262
Elsewhere in school	3.36	3.00	32.96	0.001	0.017
On the way to school/to home	2.99	2.26	135.53	0.001	0.065
Outside school	3.04	2.45	87.90	0.001	0.043
Rumor spreading/note-sending [Wilks’ Lambda = 0.553, *F*(5, 1951) = 316.01, *p* < 0.001, ŋ*_p_*^2^ = 0.447]					
Classroom	2.76	3.93	442.98	0.001	0.185
Playground	3.73	2.30	570.99	0.001	0.226
Elsewhere in school	3.51	3.51	0.02	Not sig.	0.000
On the way to school/to home	3.32	3.03	14.95	0.001	0.008
Outside school	3.26	2.90	28.46	0.001	0.014

**Table 2:** Main effects of nationality on likely aggressor for different forms of bullying/ijime.

Aggressor	Means		*F* Value (*df* = 11,965)	*p* Value	Effect Size (ŋ*_p_*^2^)
	English	Japanese			
Physical aggression [Wilks’ Lambda = 0.817, *F*(3, 1953) = 145.76, *p* < 0.001, ŋ*_p_*^2^ = 0.183]					
Pupils in the same class	2.56	3.37	202.69	0.001	0.094
Pupils in different classes	3.17	3.17	.09	Not sig.	0.000
Pupils in higher years	3.20	2.38	184.52	0.001	0.086
Verbal aggression [Wilks’ Lambda = 0.664, *F*(3, 1953) = 329.24, *p* < 0.001, ŋ*_p_*^2^ = 0.336]					
Pupils in the same class	2.54	3.89	638.69	0.001	0.246
Pupils in different classes	3.15	3.51	54.44	0.001	0.027
Pupils in higher years	3.14	2.25	233.36	0.001	0.107
Ignoring [Wilks’ Lambda = 0.647, *F*(3, 1953) = 354.89, *p* < 0.001, ŋ*_p_*^2^ = 0.353]					
Pupils in the same class	2.46	3.91	763.29	0.001	0.281
Pupils in different classes	2.93	3.37	81.44	0.001	0.040
Pupils in higher years	2.85	2.09	176.32	0.001	0.083
Social exclusion [Wilks’ Lambda = 0.729, *F*(3, 1953) = 242.47, *p* < 0.001, ŋ*_p_*^2^ = 0.271]					
Pupils in the same class	2.56	3.68	403.07	0.001	0.171
Pupils in different classes	2.94	3.38	92.65	0.001	0.045
Pupils in higher years	2.79	1.97	222.61	0.001	0.102
Stealing/hiding/taking money or belongings [Wilks’ Lambda = 0.738, *F*(3, 1953) = 231.00, *p* < 0.001, ŋ*_p_*^2^ = 0.262]					
Pupils in the same class	2.44	3.65	504.08	0.001	0.205
Pupils in different classes	2.88	3.30	74.42	0.001	0.037
Pupils in higher years	2.99	2.23	134.08	0.001	0.064
Rumor spreading/note-sending [Wilks’ Lambda = 0.698, *F*(3, 1953) = 282.17, *p* < 0.001, ŋ*_p_*^2^ = 0.302]					
Pupils in the same class	2.60	3.90	621.45	0.001	0.241
Pupils in different classes	3.08	3.64	128.00	0.001	0.061
Pupils in higher years	2.96	2.31	108.57	0.001	0.053

**Table 3:** Main effects of nationality on likely relationships between aggressor(s) and the victim for different forms of bullying/ijime.

Relationships	Means		*F* Value (*df* = 11,965)	*p* Value	Effect Size (ŋ*_p_*^2^)
	English	Japanese			
Physical aggression [Wilks’ Lambda = 0.737, *F*(3, 1953) = 282.89, *p* < 0.001, ŋ*_p_*^2^ = 0.263]					
Friends	2.05	3.19	425.06	0.001	0.179
Known but not friends	3.29	3.01	34.88	0.001	0.018
Not know very well/at all	2.99	1.94	350.99	0.001	0.152
Verbal aggression [Wilks’ Lambda = 0.669, *F*(3, 1953) = 322.53, *p* < 0.001, ŋ*_p_*^2^ = 0.331]					
Friends	2.18	3.60	655.14	0.001	0.251
Known but not friends	3.20	3.12	2.02	Not sig.	0.001
Not know very well/at all	3.00	1.97	340.31	0.001	0.148
Ignoring [Wilks’ Lambda = 0.745, *F*(3, 1953) = 222.68, *p* < 0.001, ŋ*_p_*^2^ = 0.255]					
Friends	2.24	3.42	427.42	0.001	0.179
Known but not friends	3.11	3.19	1.98	Not sig.	0.001
Not know very well/at all	2.92	2.02	271.36	0.001	0.122
Social exclusion [Wilks’ Lambda = 0.750, *F*(3, 1953) = 217.31, *p* < 0.001, ŋ*_p_*^2^ = 0.250]					
Friends	2.35	3.44	380.50	0.001	0.163
Known but not friends	3.05	3.07	0.45	Not sig.	0.000
Not know very well/at all	2.89	1.98	270.89	0.001	0.122
Stealing/hiding/taking money or belongings [Wilks’ Lambda = 0.766, *F*(3, 1953) = 199.37, *p* < 0.001, ŋ*_p_*^2^ = 0.234]					
Friends	2.20	3.30	381.91	0.001	0.163
Known but not friends	3.15	3.13	0.01	Not sig.	0.000
Not know very well/at all	3.03	2.15	215.54	0.001	0.100
Rumor spreading/note-taking [Wilks’ Lambda = 0.741, *F*(3, 1953) = 227.37, *p* < 0.001, ŋ*_p_*^2^ = 0.259]					
Friends	2.33	3.55	477.99	0.001	0.196
Known but not friends	3.22	3.35	6.53	[0.05]	0.003
Not know very well/at all	2.99	2.21	164.49	0.001	0.078

**Table 4:** Mean numbers of friends in the same class, different class in the same year group, and different year groups (SD in brackets).

	Means		*F* Value (*df* = 11,575)	*p* Value	Effect Size (ŋ*_p_*^2^)
	English	Japanese			
Number of Friends [Wilks’ Lambda = 0.827, *F*(3, 1573) = 109.54, *p* < 0.001, ŋ*_p_*^2^ = 0.173]					
Same class	6.54 (3.81)	8.16 (4.48)	67.83	0.001	0.041
Different class in the same year group	8.12 (5.47)	9.47 (5.43)	19.70	0.001	0.012
Different year groups	5.24 (3.66)	2.98 (3.24)	156.60	0.001	0.090

**Table 5:** Main effects of nationality on time spent with friends in the same class, friends in different classes but the same year group, and friends in different year groups.

Places	Mean		*F* Value (*df* = 11,965)	*p* Value	Effect Size (ŋ*_p_*^2^)
	England	Japan			
Same class [Wilks’ Lambda = 0.428, *F*(5, 1951) = 521.42, *p* < 0.001, ŋ*_p_*^2^ = 0.572]					
Classroom	3.76	4.08	19.98	0.001	0.010
Playground	4.18	1.67	2277.69	0.001	0.538
Other places in school	3.59	3.39	11.95	0.001	0.006
On the way to school/to home	3.10	3.11	0.15	Not sig.	0.000
Outside school	3.04	2.55	106.95	0.001	0.052
Different classes in same year group [Wilks’ Lambda = 0.516, *F*(6, 1950) = 304.70, *p* < 0.001, ŋ*_p_*^2^ = 0.484]					
Classroom	2.73	2.18	89.08	0.001	0.044
Friends’ classroom	2.54	2.09	64.91	0.001	0.032
Playground	3.56	1.51	1487.36	0.001	0.432
Other places in school	2.96	2.94	2.74	Not sig.	0.001
On the way to school/to home	2.77	3.35	76.77	0.001	0.038
Outside school	2.85	2.72	14.44	0.001	0.007
Different year groups [Wilks’ Lambda = 0.644, *F*(6, 1950) = 179.62, *p* < 0.001, ŋ*_p_*^2^ = 0.356]					
Classroom	1.86	1.15	321.54	0.001	0.141
Friends’ classroom	1.78	1.14	281.03	0.001	0.126
Playground	2.47	1.14	872.08	0.001	0.308
Other places in school	2.13	1.46	231.06	0.001	0.106
On the way to school/to home	2.39	1.50	317.13	0.001	0.140
Outside school	2.58	1.49	515.59	0.001	0.209

## Data Availability

The data presented in this study are available on request from the corresponding author.

## Endnote

This study was originally conducted as part of the first author’s doctoral research and published in his doctoral thesis (Kanetsuna, 2004) and, in part, in a Japanese university bulletin (Kanetsuna, 2009). However, since the bulletin article was written in Japanese and is difficult to reach an international audience, and, more importantly, since the problem of bullying/ijime continues to be a serious problem all over the world, we believe that this article will be of interest and useful to a wider audience. Therefore, we decided to republish it as an English translated version with some expansion. The bulletin article includes the results from Table 1, Table 4, and Table 5 in this article. The results shown in Table 2 and Table 3 are new for this article. Some summary findings of this article were cited briefly in Kanetsuna (2016). Comparisons between English bullying and Japanese ijime, in Smith, P.K., Kwak, K., & Toda, Y. (Eds.) (2016). School bullying in different cultures: Eastern and Western perspectives (pp. 153–169), Cambridge, U.K.: Cambridge University Press, as “Kanetsuna and Smith (submitted)”. In the present article we report the number of friends children have in their own class, in different classes, and in different year groups, and the places where they spent time with these friends; in the book chapter, a brief summary of these results was given in the text (pp. 162–163) with very limited descriptive figures but no tables or results of statistical analysis. There are no other publications using the same data.
